# Is the World Satisfied with the Medical Profession?

**Published:** 1888-06

**Authors:** L. W. Cocke

**Affiliations:** San Marcos


### IS THE WORLD SATISFIED WITH THE MEDICAL PROFESSION?

*By L. W. Cocke, M. D., San Marcos.*

Read before the A. D. M. S., at Austin, March 8, and referred to the Publishing Committee, and published in the Official Organ, by vote of the Committee.

For Daniel's Texas Medical Journal.

THE subject under consideration would not, at a cursory glance, seem to be of enough importance to the student of medicine to warrant a thesis; yet on a more profound investigation, its value and significance to the profession become more and more apparent. Indeed, it is one that should receive a careful consideration from each and every member of the profession who has the welfare of science and humanity at heart. As science advances, so, in a corresponding rate, is the human race advanced, enlightened and benefited. And verily, the progress or decline of medicine is dependent on the esteem and reverence with which the profession is held by that most conversent majority of mankink dubbed the laiety.

Hence, when the world conceives false ideas, and entertains impressions at variance with the truth in regard to our profession, it then becomes our duty to ourselves and to humanity to strive with our utmost energy to correct these falsities, and place ourselves in a true light to the world, and thereby be enabled to carry out the democratic principles of our order, doing the most good to the greatest number of people. As an example of one of the many falsely conceived ideas that the laiety hold in regard to us, is that ninety-nine men out of one hundred impute mercenary motives to us when we refuse to prescribe some patent nostrum for them, that has been judiciously advertised by the press. We are too prone to dismiss them with an ignorant protest, instead of explaining to them that they would hold us accountable for an action of an unknown drug.

It was Dr. Holmes, I believe, who makes one of his literary characters in one of his works say that "nothing is of more interest to men of science than facts, except theories." It may be pertinent to remark, at the outset, that I shall not present for your consideration any carefully constructed theories, no positive opinions in regard to the specific action of any drug, no itimized list of experiments, no new methods as to the shortest, quickest and most accessible route to reduction of heresy, as you have no doubt had an ample amount of these to test your ability. Nor, for the present, need you reasonably expect it from me under this head. The thoughts I shall present to you this evening, will perhaps be not new to you in the least. They are such thoughts and such observations as will naturally come to each and every active member of the profession who possesses a tithe of the godlike attribute of Abou Ben Adhem of old, to whom an angel came one night, in a dream, bearing in his hand the Lamb's Book of Life, with the names of all the blest, who had been washed in the Blood of the Lamb, but his name was not on the list. As the angel turned sadly away, Ben Adhem spoke, and said, "Put me down, then, as one who loves his fellow-men." Next night the angel came again, and Lo! the name of Abou Ben Adham was head of all the list.

The thought contained herein may be common to you all, but if I can only succeed in impressing one idea on any one of you that will cause a renewal of energy and a determination to work more for the advancement of the profession to that higher and loftier eminence where it justly belongs, I shall feel that my life has not been in vain.

That the interrogation contained in the headlines of this subject you have doubtless all answered in the affirmative ere this, that the dissatisfaction arising with the profession from the laiety is greater than that of any other profession, science or art, you will only have to refer to your individual experience and admit; that the causes and basis of the existing dissatisfaction is rather in the false conceits of the laiety themselves, and with the impositions that our potent and sapient lawmakers have allowed us to suffer from without one congressional or legislative voice dissenting, you will doubtless also admit, and furthermore, that there are grave reasons existing within the true bounds of the profession, that have a marked tendency to increase dissatisfaction, I shall attempt to prove in this paper. But we are pleased to see these causes being removed, one by one, by the systematic work of the medical men, the world over, as a body politic.

Man is naturally more interested in that which concerns his present welfare, health and happiness than any thing else, hence, being more dependent on the science of medicine for his physical comfort and enjoyment, and as the success and failures of that science interest him more vitally, he naturally commends and condemns it accordingly. And his abuse or praise depends more on the condition of his liver, lungs or stomach, than on any systematic course of philosophy and reasoning.

Many teachers of mental and moral science, and among them Sir William Hamilton, claim that no mind, however educated, is altogether void of superstition, and it does seem to the casual observer that the human mind is more apt to be superstitious in regard to medicine than to any other science under the sun, and the consecutive line of causes can be traced from age to age, down to the present time. From medical history, we learn that in the early periods of the world, physics was an art that was supposed to be most mysterious, and those who practiced it were supposed to hold communication with the spirit world. The belief that human beings could obtain the power of conferring good and inflicting evil on their fellow creatures, is of greater antiquity, and by its extensive prevalence, it is evident that the human mind is a soil well adapted for its reception and cultivation. Life has so many evils that the mind cannot prevent nor avert, encourages so many hopes which all ages are anxious to realize, that we cannot be astonished to find so many become the willing prey of imposters. Even the lights of Divine revelations, and being discountenanced by civil and Ecclesiastical laws, have never prevented frauds and absurdities from being encouraged. Their foundation seems to lie deep in the human heart's anxiety about futurity, its impatience for good greater than it enjoys, and its restless curiosity to penetrate the unknown and meddle with the forbidden. Have you ever paused to consider how much superstition the enlightened children of the nineteenth century have inherited from antiquity? how it's helping to clog the wheels of science to-day, and pervert the truth from its rightful channels? We have the evidence of superstitous frauds and imposters before our eyes, constantly knocking with brazen effrontry at the very doors of science, and clamoring for admittance. The medical impostor of the dark ages simply threatened evil that he had no power to accomplish; the imposter of to-day promises good, and accomplishes evil.

The imposter of antiquity deluded the ignorant by fear and threats; the imposter of to-day flatters our knowing vanity, by explaining to us in a nutshell, and with half a column of testimonials, in the largest dailies, the whole secret of the vital principle of life, and places the Elixir of Life in our hands, at the moderate cost of $1 per bottle. The one peopled the air with wood-nymphs and water-sprites that came at his call to work evil or good, the other peoples our graveyards with the ghosts of his advocates.

By far the most potent factor that exists among the numerous causes of dissatisfaction existing with the profession to-day, originated under the protecting wing and shadows of the great Goddess of Liberty, where sit our wise and patriotic law-makers, and give the charlatan, imposter and avaricious quack the perfect liberty to carry the prescription given by his honest old family doctor, for the alleviation of some pain, to the door of the patent office, which stamps it with the State Seal of Uncle Sam's dominion, and under the name of some wonderful panacea that cures all ills that human flesh is heir to, he sallies forth, *sans reproche* with his paint brush and printer's ink, he spreads its flaming virtues on every wayside plank from Maine to Mexico, till every son of Adam and daughter of Eve, and the balance of mankind, who are suffering from some incurable malady, hears of, or sees it, and finally buys and tries it, and casts it aside, or it casts him aside, and takes up the next one, and so on, and the charlatan grows fat and sleek, with the great American Flag of Protective Tariff watching and guarding his secrets. And Uncle Sam continues, year by year, to offer such rewards for villany, to discountenance science, and to blaspheme and slander the votaries of truth, until this fair earth of ours is covered, from pole to circumference, with advertisements of patent nostrums.

There is no place under the sun where you will not meet the evidences of their enterprise. You may walk in your garden at early morn, to meditate on the mutability of human life, and you will discover that some patent mecicine man has invaded the sacred recesses of home, and painted letters, "St. Jacob's Oil," across the very gate-post of your dwelling. Yea, verily, if you take wings of the morning and fly away to the Swelling Orient, hoping to escape from the patent medicine man, you will find yourself wondering how "Green's August Flower" came to be painted, in box-car letters, over the entrance of a Bhuddist Temple.

The imposter and charlatan is no sloth. He watches closely the pulsations of science, and when it tends in a certain direction he changes his tactics and names his compounds accordingly. The genius of Pasteur discovers that the source of trouble that bids fair to rob France of her great silk industry is a microbe. His experiments for their destruction succeed, and France is saved. The gigantic brain of Radam immediately discovers that S.O. 4 will kill bugs. Has read that the human body is full of animalculæ; good for goose, good for gander, makes known the discovery; immediately the hypochondriacal Granger and historic Cowboy, who have been well rifled with the essence of the moonlight-still, conceives the idea that he's got bugs, and hastens to fill his jug this time with the Microbe Killer, and he is saved, restored to life and health, and calculates on reaching the illustrious age of "She" with the aid of Microbe Killer.

These charlatans and magnetic healers may not know the difference between Attfield's chemistry and Roth's history of Texas, but they do know that the average American will patronize anything that savors of mystery and humbug.

These have done more to make the world dissatisfied with medicine than all else combined ; done more to lower the standard of the profession ; done more to drag science down deep into the mire of degradation, along with hypocrisy and de ceit and falsehood than all the false themes that have been conceived and abandoned by men of science since the days of Hippocrates. But I am glad to say that patent nostrums have almost run their day ; they will soon be among the things of the past. Ask your druggist how much the ratio of these things is decreasing, and you will be astonished at his sales, compared with the amounts of ten years since.

Among the remaining causes that have exerted a marked influence in creating dissatisfaction, is the number of schools that have arisen and flourished from time to time, that have been based on irrational foundations, but each in its turn has had its quota of enthusiastic adherents who have cried down the *allopaths,* and denounced them as old fogies, until, at last, the remnant that remains is scarcely worthy of notice. Indeed the best policy is to let them severely alone and they are soon numbered with the things of the past. The Hydropath, if he exist at all, is perhaps safely stowed away in some museum of antiquity. The Eclectic, when he is decoyed into revealing his identity, is the first one generally to prescribe mercury, and the last and least of the sects are our brethren who are trying to make the voyage of life in the little boat that has for its motto this strange device : *Similia similibus curantur.* And the statistical reports show that they are rapidly diminishing and soon will be numbered among the things that were, and "should not ought to have been," and a few years longer they too will only be known as a strange set of fanatics, that believed or professed to believe an absolute contradiction.

The next class I desire to call your attention to is happily but seldom found in the West and more especially in our Lone Star State, unless he be a connection of some patent medicine company, is the pur-blind specialist, who attributes all the ills of humanity to the derangements of the organ which he has made his special study. It would be the height of folly to deny that all the advances in medicine are not the results of clinical observation and experimentation, combined with the thorough investigation of the functions of the different organs of the body, and the manner in which these functions are modified in health and disease by various therapeutic agents, but no real addition to our knowledge comes from the class of specialists. He is the bane of modern medicine, and the antidote is to let him severely alone. Emboldened by the tolerance with which he is being received by the profession, his arrogance assumes that all medical knowledge and wisdom is centered in his person, and that he is the Dictator, instead of a supernumerary to the profession. I trust that this delusion will soon be dispelled and that the physician of this country will arouse to the necessity of checking further growth of this excresence of this body politic.

And now among the last, yet not the least evil that exerts its influence on the welfare of professional advancements, and encourages the world at large to grow dissatisfied with medical men, is the lack of harmony and unison that exists in the profession. This lack of harmony is becoming more and more marked, even in prescribing the commonest remedies, and it stands as a reproach to your high scientific pretentions. There is a touch of sarcasm in Chas. Kingsley's Water Babes, that will bear quoting; of one case he says: "That all the doctors were called on for a report, and, of course, each one contradicted the other, else of what use was there in having men of science."

Two of the most celebrated cases that have been bulletined by the press, and have added more to the existing dissatisfaction, have been the cases of Garfield and the Crown Prince of Germany. It seems strange, indeed, to the world, that such men of science should differ so widely in their opinions of the cases, and doubtless these two case have done more to make the world think that the science of medicine is yet but blind experiment, than any other two causes on record.

And now, finally, how shall we remedy these evils? How lessen the gulf that stands between the profession and the laity? Shall every doctor be a teacher and educate the populace to a certain extent and teach them the falacies and delusions under which they labor? I doubt seriously if that would not increase the evil. We are told a "little learning is a dangerous thing," and I doubt not but you will find it so under the circumstances.

But there are existing evils that we can correct and overcome. If every member of this association will pledge himself to do his utmost, when occassion occurs, to combat the lack of harmony and unison among themselves, even as to exercise his spirit of sacrifice and presribe sulph. morph., instead of tr. opii or bromidia, rather than to publish to the world that two cannot agree, the world and the profession will reap the benefit of your self-sacrifice. If we will but exert half the energy we display in electing our aldermen, member of Congress or governor, to having some wholesome laws passed to regulate the practice of medicine, or the dispensing of drugs, by the next assembly that convenes in yonder most magnificent edifice, the Texas Capital, we will have such a system of laws that will far excel any of our sister States, that men of high professional worth will seek refuge under the banner of our grand and glorious Lone Star, till no part of the universe will excell us in the science of medicine. Let us unite as one man, and at the Galveston meeting make it known, that we will support no man, from Senator down to the private secretary of the mayor, unless he declare for reform and high tariff for medicine, and low tariff for everything else.

